# Cetacean Morbillivirus and *Toxoplasma gondii* Co-infection in Mediterranean Monk Seal Pup, Italy

**DOI:** 10.3201/eid2704.204131

**Published:** 2021-04

**Authors:** Antonio Petrella, Sandro Mazzariol, Iolanda Padalino, Gabriella Di Francesco, Cristina Casalone, Carla Grattarola, Giovanni Di Guardo, Camilla Smoglica, Cinzia Centelleghe, Claudia Gili

**Affiliations:** Istituto Zooprofilattico Sperimentale della Puglia e della Basilicata, Foggia, Italy (A. Petrella, I. Paladino);; University of Padova, Padua, Italy (S. Mazzariol, C. Centelleghe);; Istituto Zooprofilattico Sperimentale dell'Abruzzo e del Molise, Teramo, Italy (G. Di Francesco);; Istituto Zooprofilattico Sperimentale del Piemonte, Liguria e Valle d’Aosta, Turin, Italy (C. Casalone, C. Grattarola);; University of Teramo Faculty of Veterinary Medicine, Teramo (G. Di Guardo, C. Smoglica);; Stazione Zoologica Anton Dohrn, Naples, Italy (C. Gili)

**Keywords:** cetacean morbillivirus, *Toxoplasma gondii*, Mediterranean monk seal, *Monachus monachus*, pinniped, co-infection, pathology, microbial pathogenesis, viruses, Italy

## Abstract

A Mediterranean monk seal (*Monachus monachus*) pup from the southern Adriatic coast of Italy showed cetacean morbillivirus (CeMV) and disseminated *Toxoplasma gondii* co-infection, which probably resulted from CeMV-induced immunosuppression. These findings are of concern for the conservation of this critically endangered species.

The Mediterranean monk seal (*Monachus monachus*), the most rarely occurring pinniped worldwide, ranks among the most endangered marine mammal species. A few breeding colonies remain along the shores of Greece, Turkey, and Cyprus as well as in Atlantic waters close to Cabo Blanco, Mauritania, and Madeira (*1*).

Monk seals are deemed to be officially extinct in many countries, including Italy. A monk seal pup was found alive along the southern Adriatic coast of Italy; it died after rehabilitation attempts. We performed a detailed necropsy on January 28, 2020, within 12 hours after death. Postmortem examination confirmed the animal was a female weaning pup; it had a poor body condition score. During necropsy, we collected samples from the animal’s brain, spinal cord, lungs, liver, kidneys, lymph nodes, spleen, intestine, muscles, and tonsils for biomolecular analyses against viral and nonviral pathogens, with special emphasis on cetacean morbillivirus (CeMV) (*2*,*3*) and *Toxoplasma gondii* (*4*) (Appendix). We fixed all the tissue samples promptly in 10% neutral buffered formalin and routinely processed them for conventional histology and for morbillivirus and *T. gondii* immunohistochemistry. We used a commercially available monoclonal antibody against canine distemper virus (CDV) nucleoprotein (Veterinary Medical Research and Development, https://vmrd.com) and a rabbit polyclonal antibody against *T. gondii* (MyBioSource, https://www.mybiosource.com) (*5*,*6*).

We found extensive multifocal brain hemorrhages, most likely caused by a severe arteritis that also involved major cardiac vessels. The brain showed a multifocal, severe, nonsuppurative meningoencephalitis, closely associated with extensive and multifocal hemorrhages. We detected a diffuse, bilateral, chronic, and moderate interstitial pneumonia associated with a marked bronchiolar epithelial hyperplasia; we observed positive immunohistochemistry labeling for morbilliviral antigen within hyperplastic epithelial cells (Figure). Round, variably sized protozoan cysts positively stained with the *T. gondii* antibody were visible in the lung, within myocardial inflammatory foci, and in the tunica media of the aorta and pulmonary vessels. Lymphoid tissues exhibited a widespread and severe immune cell depletion.

**Figure:**
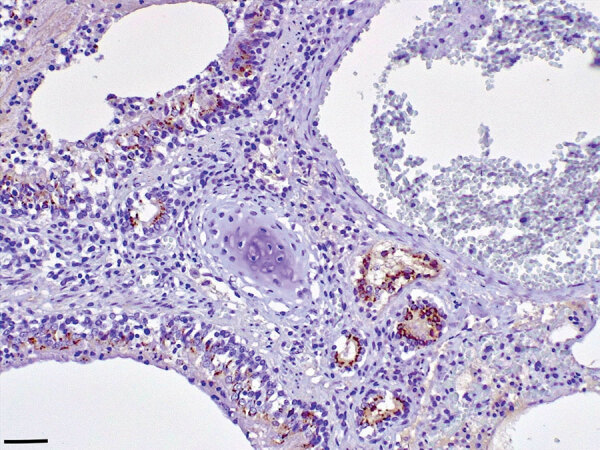
Lung tissue from a Mediterranean monk seal pup that died shortly after it was found along the southern Adriatic coast of Italy, showing positive immunostaining for morbillivirus antigen in bronchial/bronchiolar and alveodar epithelial cells, both normal and hyperplastic. Immunohistochemical analysis using an antibody against the nucleoprotein antigen of canine distemper virus (1:100 dilution), Mayer hematoxylin counterstained. Scale bar indicates 100 μm.

Through biomolecular analyses (*2*,*3*), we detected CeMV genetic fragments in brain, lung, and spleen tissues preserved in RNAlater solution (ThermoFisher, https://www.thermofisher.com) and frozen lung tissue. Fragments showed a strong homology with a CeMV isolate (complete genome GenBank accession no. MH430938.1): the brain fragment (GenBank accession no. MW266078) was 397 bp long and was 98.25% homologous; the lung fragment (GenBank accession no. MW266077), 402 bp long, was 98.5% homologous; and the spleen fragment (GenBank accession no. MW266079), 152 bp long, was 99.3% homologous. In addition, we detected biomolecular positivity for *T. gondii* in skeletal muscle and lymph nodes, which supports immunohistochemical evidence.

Co-infections by morbilliviruses and *T. gondii* are well known among terrestrial and aquatic mammals, yet they have been rarely described in pinnipeds. Seals are known to be susceptible to CDV as well as to phocine distemper virus (*7*); CeMV infection has also been reported in monk and harbor seals (*Phoca vitulina*) (*6*). In 1997, half of the Mediterranean monk seals inhabiting the shores of Mauritania died and were found to have been infected with a CeMV-like agent; a similar virus was subsequently identified in a few monk seals from Greek waters (*6*). The cause of the die-off in 1997 remains unclear; biotoxins were also detected in dead seals (*8*).

The meningoencephalitic and pneumonic lesions found in the monk seal we investigated could also be associated with severe infection by *T. gondii.* Indeed, *T. gondii*–associated deaths have been reported as a significant threat to the health and conservation of Hawaiian monk seals (*Neomonachus schauinslandii*) (*9*). In the Mediterranean region, no similar cases have been previously reported other than in cetaceans, in which *T. gondii* has been recognized as a possible cause of death either alone or in association with CeMV (*6*). The young age of this monk seal suggests that CeMV or *T. gondii* infections could have been vertically acquired; the range of the severity and chronicity of *T. gondii*–associated lesions further suggest a prolonged persistence of the protozoan agent in the animal’s circulation.

Previous *T. gondii* infection seems a plausible explanation for a subsequently acquired CeMV infection causing immunosuppression that led to disseminated toxoplasmosis. Nevertheless, we cannot exclude the possibility that CeMV acted as a primary pathogen. Previous reports of CeMV in Hawaiian monk seals, coupled with putative vertical transmission of *T. gondii,* indicate the need for careful evaluation of *T. gondii* and CeMV as potential threats to the health and conservation of Mediterranean monk seals. We recommend adequate and thorough seroepidemiologic and postmortem pathologic surveillance to assess the real risk posed by these 2 pathogens (*10*). An ad hoc infectious risk analysis protocol would enable investigators to address CeMV and *T. gondii* infections either separately or in combination by developing specific immunization protocols, such as those successfully employed on the Hawaiian monk seal population.

Appendix Additional information about cetacean morbillivirus and Toxoplasma gondii co-infection in Mediterranean monk seal pup.
