# Supplementary material for: Cetacean Morbillivirus and Toxoplasma gondii Co-infection in Mediterranean Monk Seal Pup, Italy
Source: Emerg Infect Dis. 2021 Apr;27(4):1237–9. doi: 10.3201/eid2704.204131 (PMC8007312; doi:10.3201/eid2704.204131)
Supplement: Appendix — Additional information about cetacean morbillivirus and Toxoplasma gondii co-infection in Mediterranean monk seal pup. [file 20-4131-Techapp-s1.pdf]

Article DOI: https://doi.org/10.3201/eid2704.204131

Cetacean Morbillivirus and *Toxoplasma gondii* Co-Infection in Mediterranean Monk Seal Pup, Italy

Appendix

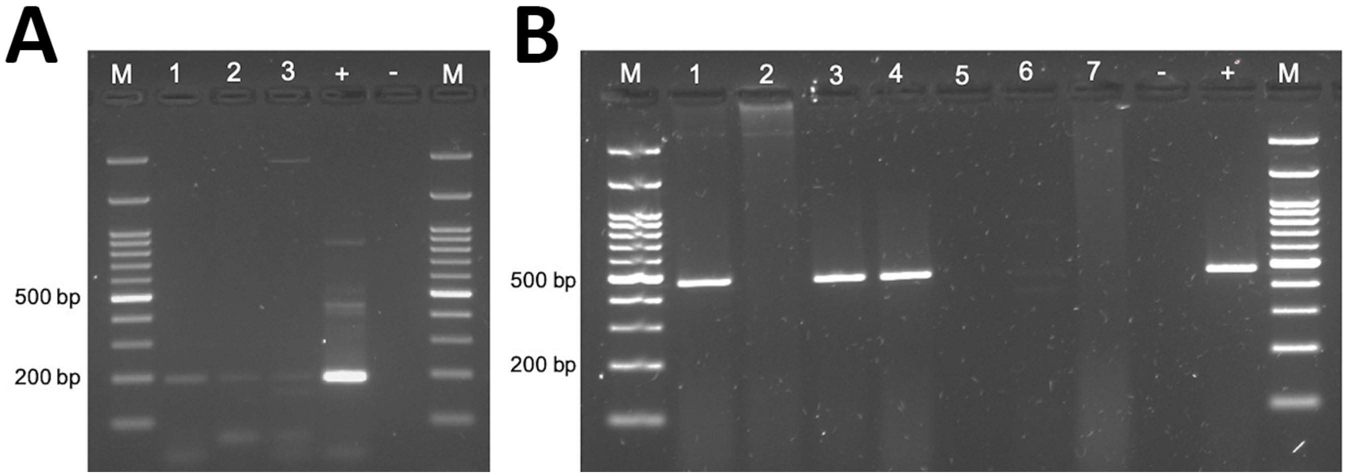

Appendix Figure. Agarose gel results of the PCR protocols used for cetacean morbillivirus molecular detection in Mediterranean monk seal tissues. A) Results of the nested reverse transcription PCR using the DMV–13F and DMV–13R primer pairs. Fragment length 200 bp (Centelleghe et al. [*2*]). M, DNA marker, 100 bp; lane 1, lung collected in RNA-later stabilization solution; lane 2, brain collected in RNA-later stabilization solution; lane 3, spleen collected in RNA-later stabilization solution (GenBank accession no. MW266078); +, PCR positive control; −, PCR negative control. B) Results of conventional reverse transcription PCR using the DMV–1F and DMV–1R primer pairs. Fragment length: 474 bp. (Beffagna et al. [*3*]). Lane 1, lung collected in RNA-later stabilization solution (GenBank accession no. MW266077); lane 2, spleen collected in RNA-later stabilization solution; lane 3, brain collected in RNA-later stabilization solution (GenBank accession no. MW266078); lane 4, lung collected frozen; lane 5, spleen collected frozen; lane 6, intestine collected frozen; lane 7, liver collected frozen; −, PCR negative control; +, PCR positive control.
